# A New Insight into 6-Pentyl-2H-pyran-2-one against *Peronophythora litchii* via TOR Pathway

**DOI:** 10.3390/jof9080863

**Published:** 2023-08-21

**Authors:** Yinggu Wu, Xinyu Li, Li Dong, Tong Liu, Zhengbin Tang, Runmao Lin, Justice Norvienyeku, Mengyu Xing

**Affiliations:** 1 Key Laboratory of Green Prevention and Control of Tropical Diseases and Pests, Ministry of Education, School of Tropical Agriculture and Forestry, Hainan University, Haikou 570228, China; wyg123452021@163.com (Y.W.); 18238536733@163.com (X.L.); zakkizz@163.com (L.D.); liutongamy@sina.com (T.L.); rudytang@aliyun.com (Z.T.); linrm2010@163.com (R.L.); 2 Sanya Nanfan Research Institute, Hainan University, Sanya 572025, China

**Keywords:** *P. litchii*, 6-pentyl-2H-pyran-2-one (6PP), TOR pathway, CRISPR/Cas 9, *PlYY1*

## Abstract

The litchi downy blight disease of litchi caused by *Peronophythora litchii* accounts for severe losses in the field and during storage. While ample quantitative studies have shown that 6-pentyl-2H-pyran-2-one (6PP) possesses antifungal activities against multiple plant pathogenic fungi, the regulatory mechanisms of 6PP-mediated inhibition of fungal pathogenesis and growth are still unknown. Here, we investigated the potential molecular targets of 6PP in the phytopathogenic oomycetes *P. litchii* through integrated deployment of RNA-sequencing, functional genetics, and biochemical techniques to investigate the regulatory effects of 6PP against *P. litchii*. Previously we demonstrated that 6PP exerted significant oomyticidal activities. Also, comparative transcriptomic evaluation of *P. litchii* strains treated with 6PP Revealed significant up-regulations in the expression profile of TOR pathway-related genes, including *PlCytochrome C* and the transcription factors *PlYY1*. We also noticed that 6PP treatment down-regulated putative negative regulatory genes of the TOR pathway, including *PlSpm1* and *PlrhoH12* in *P. litchii*. Protein-ligand binding analyses revealed stable affinities between PlYY1, PlCytochrome C, PlSpm1, PlrhoH12 proteins, and the 6PP ligand. Phenotypic characterization of *PlYY1* targeted gene deletion strains generated in this study using CRISPR/Cas9 and homologous recombination strategies significantly reduced the vegetative growth, sporangium, encystment, zoospore release, and pathogenicity of *P. litchii*. These findings suggest that 6PP-mediated activation of *PlYY1* expression positively regulates TOR-related responses and significantly influences vegetative growth and the virulence of *P. litchii*. The current investigations revealed novel targets for 6PP and underscored the potential of deploying 6PP in developing management strategies for controlling the litchi downy blight pathogen.

## 1. Introduction

Litchi (*Litchi chinensis* Sonn.) is an economically important evergreen fruit tree crop cultivated across selected subtropical and tropical regions, including southern provinces of China, South Africa, Australia, Hawaii, and Israel [1]. The economic potential of litchi cultivation is not fully harnessed due to the prevalence of diseases. Reportedly, the Litchi downy blight disease caused by *P. litchii* is one of the most destructive diseases of Litchi and accounts for 20–30% onfield and post-harvest losses per year [2]. The devastating Litchi downy blight pathogen causes decay and rapid browning of the litchi fruits on the field or during the post-harvest period [3]. Management of *P. litchii* heavily relies on the application of synthetic agrochemicals [4,5]. Onfield and post-harvest application of high-efficacy synthetic chemicals is increasingly becoming a significant concern to human health, environmental safety, and biodiversity, prompting the search for microbes or plant-based environment-friendly bioactive secondary metabolites for developing fungicides/oomyticides for controlling phytopathogenic oomycetes, including *P. litchii* [6,7,8].

Previous studies have shown that 6-Pentyl-2H-Pyran-2-One (6PP, CAS:27593-23-3), a bioactive secondary metabolite isolated from *Trichoderma viride* in the 1972s, possesses antifungal activities against a broad range of phytopathogenic fungi, including *Sclerotinia sclerotiorum*, *Cronartium ribicola*, *Fusarium oxysporum*, *Fusarium moniliforme*, *Verticillium dahliae*, *Verticillium fungicola*, *Aspergillus flavus*, *Aspergillus niger*, and *Aspergillus glaucus* [9,10,11,12,13,14,15]. Additionally, we observed that treating *P. litchii* strains with 6PP significantly reduced vegetative growth, suppressed sporangia production, compromised zoospore release, and pathogenicity of *P. litchii* (unpublished data). However, the action mechanism of 6PP against *P. litchii* is unclear, and TOR pathway-related genes of response to 6PP stress in *P. litchii* are not understood.

Core cellular pathways, including the conserved Ser/Thr protein kinase target of the rapamycin (TOR) pathway, have been identified as a key regulator of cellular growth, survival, metabolism, ribosome biogenesis, translation, and transcription, processes [16,17,18]. Studies have shown that the binding of rapamycin to proteins associated with the complexes (TORC1 and TORC2) initiates signal transduction events that drive vital cellular processes. For instance, mRNA translation and autophagy-dependent proteolysis modulate microbial response to nutrient deficiency and environmental stress. Studies have shown that TORC2 influences cellular and morphological characteristics, including dynamic transformations in the actin cytoskeleton during polar growth [19,20]. Moreover, previous studies have shown that TOR is both a negative regulation factor and one of the vital regulators of autophagy [21,22,23]. Energy starvation and external stresses initiate the activation of autophagy by suppressing the activation of the TOR pathway. Instead, abundant energy induces TOR pathway activity, but autophagy activity is suppressed [24]. The TOR pathways are essential in plant pathogen fungus’s growth and pathogenicity. For example, studies have shown that TOR pathway-related genes regulate vegetative development, virulence, or deoxynivalenol (DON) production in *Botrytis cinerea*, *V. dahliae*, *F. oxysporum*, and *Fusarium graminearum* [25,26,27,28,29] and contribute to appressorium formation in the plant fungal pathogen *Magnaporthe oryzae* [30]. In *Arabidopsis*, the TOR pathway is associated with the response to sugar and the maintenance of root tip homeostasis [31]. Also, TOR pathway-dependent processes profoundly influence the development and survival of cancer cells in Humans [32]. However, the regulatory mechanisms of 6PP against *P. litchii* and its influence on essential pathways associated with microbial pathogenesis, including the TOR pathway, remain unclear.

Here, we monitored the transcriptomic dynamics in *P. litchii* treated with 6PP and identified 6PP-responsive genes, including the YinYang1 (YY1) transcription factor. Additional results from molecular docking analyses and phenotypic characterization of *YY1* gene deletion strains indicated that 6PP possibly suppresses vegetative growth, sporulation, cyst formation, and pathogenesis of *P. litchii* by regulating the activation of the TOR pathway. These findings highlight the critical role of 6PP-mediated regulation of *YY1* expressions on the TOR pathway and the impact on cellular/vegetative development and the pathogenesis of phytopathogenic oomycetes.

## 2. Materials and Methods

### 2.1. Plant Materials, Cultural Media, and Reagents

Tender litchi leaves (cv. Baitangying) were collected from a Hainan University orchard (Haikou, China). Leaf shapes and sizes were uniform and disease-free. Fresh and healthy Litchi fruits were harvested from orchards in Haikou, China, for infection assays. All fruits were healthy, uniform in shape, and at 80% ripe.

Carrot juice agar (CJA) medium was prepared by incorporating the juice from 0.2 kg of crushed carrots into the water to attain a final volume of 1 L, adding 15 g agar/L for solid media.

The 6PP was an analytical-grade standard purchased from Shanghai Aladdin Bio-Chem Technology Co., Ltd., Shanghai, China.

### 2.2. Transcriptome Analysis

Due to its volatile nature, 6PP was evaluated for its antifungal activity against *P. litchii* by air fumigation. The fumigation device is composed of two petri dish bottoms. One bottom of the petri dish contains *P. litchii*, and the other bottom of the petri dish contains 6PP, then wrapped with parafilm. The fumigation device allows air exchange, but 6PP has no direct contact with *P. litchii*. *P. litchii* was cultured on CJA medium for 3 days, and fumigated with 6PP (86 μg/mL) for another day, then the biomass was collected and frozen in liquid nitrogen. The 6PP concentration (86 μg/mL) was selected based on our preliminary results, which are the 2.0 × EC50 values of 6PP against *P. litchii* mycelial growth (Appendix A). Total RNA extraction, isolation, and purification methods were operated as the manufacturer’s protocol using the TRIzol reagent (Invitrogen, Carlsbad, CA, USA), and the RNA amount and purity, and integrity were examined using the NanoDrop ND-1000 (NanoDrop, Wilmington, DE, USA), Bioanalyzer 2100 (Agilent, Santa Clara, CA, USA) and analyzed via electrophoresis with denaturing agarose gel. A complementary DNA (cDNA) library was created by Invitrogen SuperScript™ II Reverse Transcriptase (Invitrogen, cat. 1896649, Carlsbad, CA, USA), for sequencing by Illumina Novaseq™ 6000 platform. Sequencing adapters and low-quality sequencing data were removed, and valid data were used for analysis. Genes expression in each sample was calculated by Reads Per Kilobase of the exon model per Million mapped reads (RPKM) value [33]. Four biological replicates were used.

### 2.3. Identification of TOR Pathway-Related Genes in P. litchii 

Genome (ID: PRJNA290406) and proteome (GWHAOTU00000000.1) data for *P. litchii* were downloaded from GenBank and National Genomics Data Center separately, and relative TOR pathway protein sequences reported for *F. graminearum*, *Homo sapiens*, *Saccharomyces cerevisiae*, and *M. oryzae* were obtained. Subsequently, the method of BlastP was adapted to blast and search (Total score > 0.43) in *the P. litchii* database we have established. After searching and matching to transcriptome data, TOR pathway genes regulated by 6PP were identified based on their differential expression pattern and predicted domains, molecular weight, and isoelectric points using Simple Modular Architecture Research Tool (SMART) database, NCBI Batch CD-Search (https://www.ncbi.nlm.nih.gov/Structure/bwrpsb/bwrpsb.cgi, accessed on 23 October 2021) and online program ExPASy ProtParam (https://web.expasy.org/protparam, accessed on 7 November 2022). Additionally, we deployed MEME suit for further DNA-biding motif analyses (MEME—Submission form (meme-suite.org, accessed on 7 November 2022) online website.

### 2.4. Molecular Docking

Ligand 6PP structure, obtained from PubChem database, was converted from a two-dimensional model to mol 2 format by Openbable (version 3.1.1) process. Target protein structures were required for signal peptide prediction by SignalP-4.1 (https://services.healthtech.dtu.dk./service.hph?SignalP-4.1, accessed on 22 December 2021), and then homology modeling was processed and analyzed for applicability by SWISS-MODEL (https://swissmodel.expasy.org, accessed on 22 December 2021) and SAVES v.6.0 (https://saves.mbi.ucla.edu, accessed on 22 December 2021). Furthermore, autodock software (version 1.5.6) and python software (version 2.5) was used for docking analyses using the default parameters to calculate the binding affinities between ligand and receptors, and 50 interaction models were accepted to establish with default docking parameter [34]. Interaction models were visualized by Discovery Studio software (version 4.5), and five replicates were used.

### 2.5. Transcriptional Activation Test

Transcriptional activation is a ubiquitously essential process that regulates gene modification. Additional tests were performed to examine the activation abilities of the *PlYY1* gene. By linearizing the pGBKT7 cassette with *Eco*RI and *Bam*HI sites, *the PlYY1* gene was constructed (pGBKT7-PlYY1), using primer pairs listed in Supplement Table S1. For toxicity testing, transformed yeast constructs pGBKT7 and pGBKT7-*PlYY1* were inoculated in liquid SD-Trp media and diluted into different concentration solutions to compare growth volumes (0.5, 10^−1^, 10^−2^, 10^−3^, and 10^−4^). Consecutively, pGBKT7-*PlYY1* was cultured at SD-Trp-Leu, SD-Trp-Leu-His-Ade, and SD-Trp-Leu-His-Ade+X-α-gal medium separately to ascertain whether the target vector had been transferred into yeast AH109 correctly and has a Transcriptional activation ability. In order to determine the accuracy of the experiment, positive co-transformed control pGBKT7-p53+pGADT7-ADT and negative control pGBKT7 were conducted. This experiment was performed by three times.

### 2.6. Targeted Deletion PlYY1 Gene by CRISPR/Cas 9 

To knock out the gene efficiently, two fragments of sgRNA were synthesized and cloned into an “all-in-one” pYF515 vector, respectively. Donor DNA hygromycin B resistance gene (*HPH*) and two homologous flanking sequences (about 1kb) were connected by overlap extension PCR and cloned to linearized plasmid pBluescript II KS+. According to the previous gene knock-out technique [35], All-in-one plasmid pYF515 and Homologous knock-out plasmid pBluescript II KS+ were cotransformed into protoplasts of *P. litchii*, and transformants were separated by CJA medium supplemented with G418 at concentration 20 μg/mL. The conventional cetyltrimethylammonium bromide (CTAB) method was used to extract genomic DNA. Moreover, transformants were verified and identified by polymerase chain reaction (PCR) in three rounds. Also, PCR products were sequenced if transformants identification was verified, and all primers are provided in Table S1.

### 2.7. Phenotype Analysis

For phenotypic examinations, the two independent *PlYY1* gene deletion strains (∆*PlYY1*-1 and ∆*PlYY1*-2), the wild-type, and the complementation (▲*PlYY1*-2) strains were inoculated in CJA medium at 25 °C for 5-days in darkness, and mycelial growth rate, sporangia production, and zoospore release rate were investigated. The total harvest volume of sporangia was 30 mL for each plate, and the release of zoospore was induced by low temperature (12 °C). To determine the oospores production, *P. litchii* was inoculated on CJA medium, and the 10th and 15th day, plugs (1 cm × 1 cm) were cut from 1 cm around the inoculation site, and oospores were counted under a microscope. All experiments were repeated five times, with three replicates in each experiment.

### 2.8. Pathogenicity Tests

To investigate whether the pathogenic capacity of mutants was affected, both the leaves and lychee fruits were used for testing. Three strains, WT, ∆PlYY1-1, and ∆PlYY1-2, were incubated as described in Section 2.7. Leaves were inoculated by mycelia disc (Φ = 6 mm), and fruits were inoculated with 20 mL sporangia suspension at a concentration of 1 × 10^4^ sporangia/mL. Lesion size and pattern were analyzed at 48 hpi and 60 hpi at room temperature. Results were generated from three independent biological experiments, with each experiment comprising three technical replicates. Each biological replicate consists of 27 leaves and 30 fruits. 

### 2.9. The Sensitivity Determination of PlYY1 to 6PP

Air fumigation was utilized, and both WT and mutant strains’ discs (Φ = 6 mm) were inoculated in CJA medium, which was fumigated with 6PP for 5 days at 25 ± 1 °C. The concentration of 6PP is 1.0 × EC50. Mycelial growth was measured and used in deriving the inhibition rate. Negative control (NK) was set up without 6PP. This experiment was replicated three times with five copies each time.

### 2.10. Data Analysis

Statistical computations were performed using the one-way ANOVA (Analysis of Variance) with Tukey’s multiple comparisons test, and all results exhibited mean ± SEM. Significance differences recorded between treatments and control groups are denoted with an asterisk (*). A single asterisk (*) represented *p* < 0.05, while double asterisks (**) represented *p* < 0.01, and ns represented no significant difference.

## 3. Results

### 3.1. Analysis of Differentially Expressed Genes and Homologous Proteins of TOR Pathway in P. litchii

**Figure 1:**
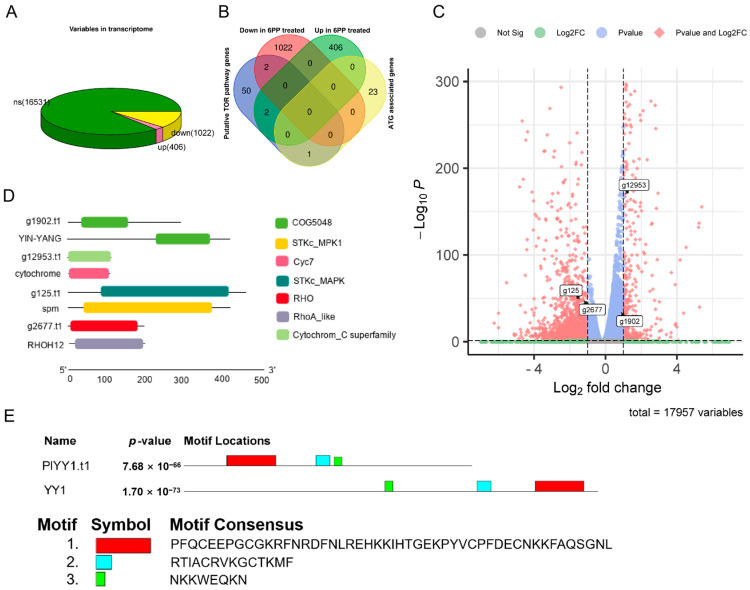
Analysis of gene expression of *P. litchii* under 6PP stress and identification of homologous proteins of TOR pathway. (**A**): differentially expressed genes. (**B**): The overall expression levels of TOR pathway genes and autophagy-related genes. (**C**): The expression levels of transcripts of four TOR pathway-related genes. (**D**): Analysis of the domain of the amino acid sequence encoded by four genes. (**E**): Comparative motif sequence of *PlYY1* coding proteins and selected orthologs protein YY1 motif. Note: ns means no significance: up means up-regulation: down means down-regulation.

Following the RPKM Recruitment Analysis, differentially expressed genes (GEGs) were identified using a threshold *p*-value < 0.05 and |log2Fold change| ≥ 1. A comprehensive analysis of 17,959 genes was conducted in the Transcriptome data, comparing the treatment and control groups. Of these, 1428 genes exhibited differential expression, with 406 genes up-regulated and 1022 genes down-regulated. (Figure 1A). This study employed the BlastP method to assess the sequence similarity in the proteome, given that the amino acid sequence is more conserved than the nucleic acid base sequence. The known TOR pathway-related protein sequences in *F. graminearum*, *H. sapiens*, *S. cerevisiae*, and *M. oryzae* were selected as search sequences to compare them in the proteome database of *P. litchii*. This comparison resulted in the identification of 55 potential proteins associated with the TOR pathway using a protein sequence similarity threshold of 30%. Additionally, 24 autophagy-related genes (ATG) were identified from *P. litchii* through homologous gene comparison. Furthermore, by conducting transcriptome differential expression gene analysis, it was observed that two TOR pathway-related genes, g1902.t1 (evalue: 8.50 × 10^−18^, identity: 57.719%) and g12953.t1 (value: 4.76 × 10^−48^, identity: 64.423%), were significantly up-regulated, while two other TOR pathway-related genes, g125.t1 (evalue: 7.39 × 10^−128^, identity: 52.632%) and g2677.t1 (value: 9.24 × 10^−71^, identity: 52.778%), were significantly down-regulated. whereas no autophagy gene was not affected (Figure 1B,C). Protein alignment revealed the YinYang1 transcription factor (YY1) as the possible ortholog of PlYY1.t1, while conserved domain analyses revealed g12953.t1, g125.t1, and g2677.t1 as putative orthologs of cytochrome C, spm1, rhoH12 in *P. litchii*. Molecular weight and isoelectric point of proteins were analyzed, and PlYY1.t1, g12953.t1, g125.t1, and g2677.t1 were calculated for 31.94949 KDa and 8.76, 12.04376 KDa and 9.36, 50.49555 KDa and 5.45, 21.97824 KDa and 7.55, respectively (Figure 1D). Furthermore, motif analysis showed a consistency motif, but there is a slight difference in the layout, and the red box motif of YY1 contains four C2H2-type zinc-finger motifs (Figure 1E), suggesting that *PlYY1 is* also coding protein with C2H2-type zinc-finger motifs and underscores the likely involvement of *PlYY1* in regulating the expression/functions of other genes. Previous studies have reported the functions of *cytochrome C*, *spm1*, and *rhoH12* in plant pathogenic fungi, but almost no report on *YY1*. Thereby, *PlYY1* was selected for further study. 

### 3.2. Binding Affinity Activity Analysis

**Figure 2:**
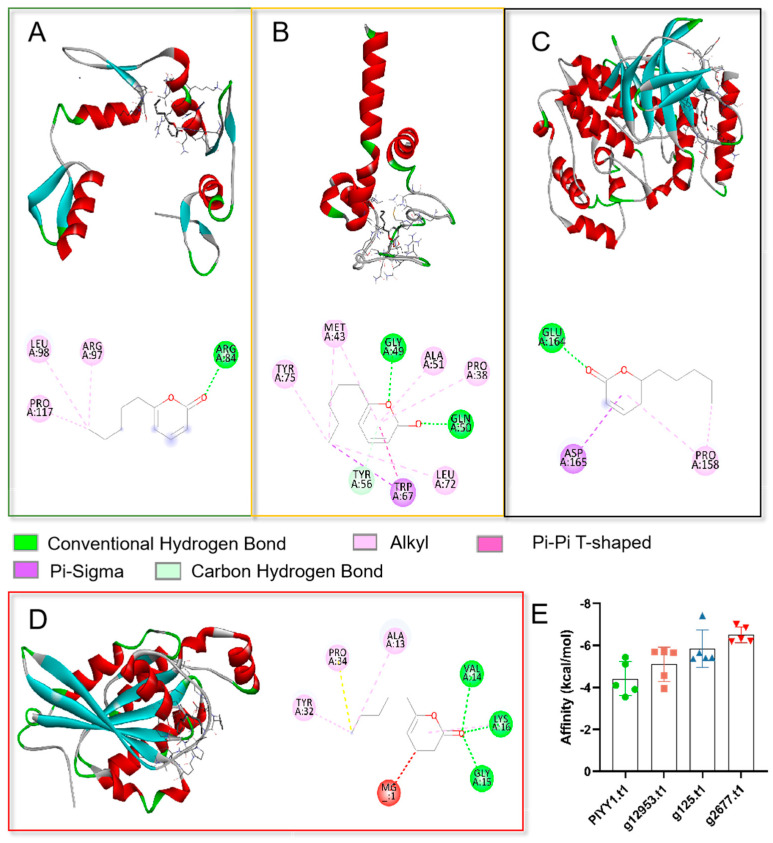
Molecular docking analysis of four proteins with ligand 6PP. (**A**): The three-dimension (above) and two-dimension (below) of the docking structure between the PlYY1.t1 protein model and 6PP. (**B**): The three-dimension (above) and two-dimension (below) of the docking between the 12953.t1 protein model and 6PP. (**C**): The three-dimension (above) and two-dimension (below) of the docking between the model of 125.t1 protein and 6PP. (**D**): The three-dimension (left) and two-dimension (right) of the docking between the 2677.t1 protein and 6PP. (**E**): Showed the binding energies recorded between the four proteins and the 6PP ligand.

To elucidate the potential action mechanism of the TOR pathway and 6PP at the molecular level, we conducted molecular docking analyses of the ligand (6PP) to the binding sites of PlYY1.t1, g12953.t1, g125.t1, and g2677.t. Our findings revealed that the main forces involved in the interactions were hydrogen bonding, van der Waals forces, and conjugate bonding (Figure 2A–D). The binding affinities of the 6PP ligand to PlYY1.t1, g12953.t1, g125.t1, and g2677.t1 were determined to be −5.43, −5.70, −7.42, and −7.0 kcal/mol, respectively, indicating a strong binding interaction (Figure 2E).

A binding energy below −5.0 kcal/mol signifies a strong binding capability, with lower binding energy implying a more stable interaction between the ligand and molecule. The outcomes of the docking analysis indicate that four proteins possess potential binding sites for 6PP.

### 3.3. Transcriptional Activation Analysis

**Figure 3:**
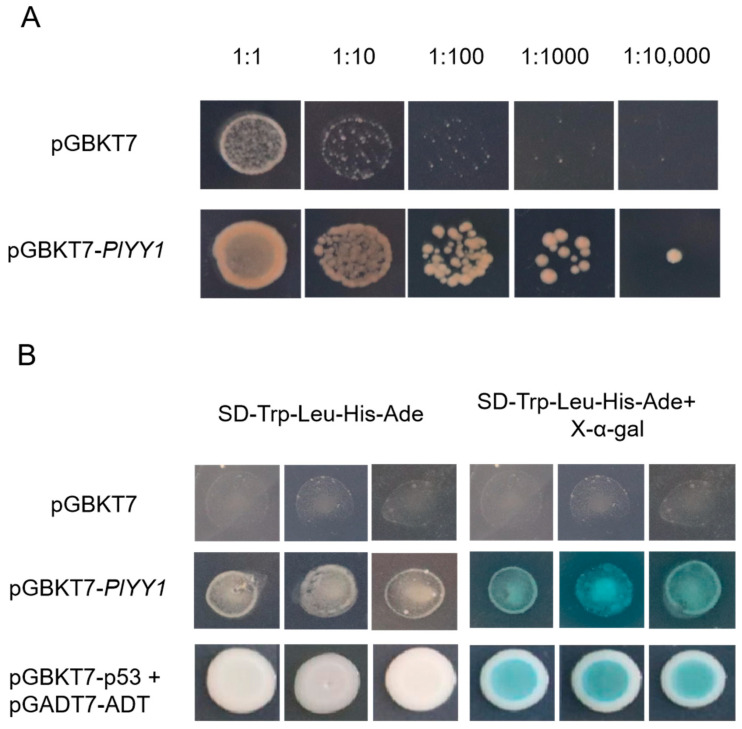
Toxicity testing and self-activation testing. (**A**): The growth condition of pGBKT7-PlYY1 construct at different dilution ratios. (**B**): Transcription activation ability test at SD-Trp-Leu-His-Ade and SD-Trp-Leu-His-Ade+X-α-gal mediums.

The results of the toxicity test conducted on pGBKT7-*PlYY1* versus control pGBKT7 indicated that the pGBKT7-*PlYY1* construct did not impact yeast growth, as the undifferentiated growth was performed in the same diluted concentration solutions on the SD-Trp medium. Meanwhile, the pGBKT7-*PlYY1* cassette had been successfully transferred to the yeast strain (Figure 3A). Furthermore, the growth phenotype and blue spots were observed in the target yeast and positive controls on the SD-Trp-Leu-His-Ade, and X-α-gal mediums, while they were absent in the negatives (Figure 3B). Thus, it can be inferred that the *PlYY1* gene possesses self-activation ability.

### 3.4. PlYY1 Is Essential for Mycelium Growth, Sporangium Production and Zoosporogenesis

**Figure 4:**
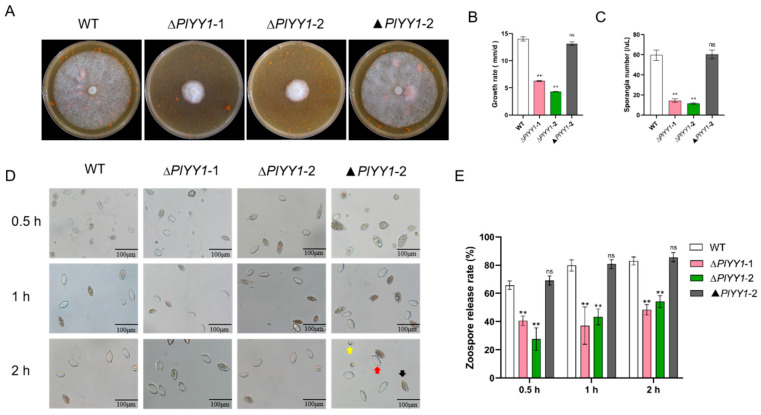
The effects of *PlYY1* on mycelium growth, sporangium formation, and zoospore release. (**A**): Colonies of four strains were cultured on CJA medium at 25 °C in the dark for 5 days. (**B**): The growth rate of four strains. (**C**): sporangia production of four strains. (**D**,**E**): Zoospore release rate of four strains. Photographs were taken at 0.5 h, 1 h, 2 h. Note: The black arrow points to the unreleased sporangia, the red arrow points to the sporangia that have released the zoospores, and the yellow arrow points to the zoospores. (** *p* < 0.01, ns represented no significant difference).

Mycelium growth, sporangial production, and zoospore release are the vegetative growth and asexual growth of *P. litchii*. Selected biological indicators, including mycelial growth rate, sporangia number, zoospore release rate, and oospore production, were monitored in wild-type and mutant strains to unravel the biological function of *the PlYY1* coding gene in *P. litchii*. The results showed significantly reduced vegetative growth and sporangia production in the *PlYY1* gene defective strains (Figure 4A–C). Zoospore release rates were observed after the sporangia suspension was incubated at 12 °C for 0.5, 1, and 2 h. The sporangia of ∆*PlYY1*-1 and ∆*PlYY1*-2 mutants exhibited a release rate of only 38.70% and 24.05% at 0.5 h, 38.27% and 44.86% at 1 h, and 45.77% and 54.66% at 2 h, respectively. In contrast, WT and ▲*PlYY1*-2 sporangia displayed a release rate of 66.13% and 68.50%, 74.72%, 80.48%, 81.82%, and 84.70% at the same time points (Figure 4D,E). Therefore, our results indicated that *PlYY1* affected the vegetative growth and asexual growth of *P. litchii*.

### 3.5. PlYY1 Has Not Affection for Oospore Development

**Figure 5:**
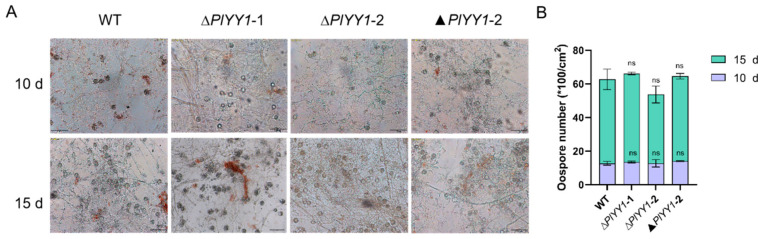
The effects of *PlYY1* on oospore production. (**A**): Morphology of oospores, photographs were taken on the 10th and 15th days. (**B**): The oospore numbers were calculated.

WT, ▲*PlYY1-2*, and *PlYY1* mutants were cultured on CJA medium for 10 or 15 days and the oospore numbers were calculated. We found that there was no significant difference among WT, ∆*PlYY1*-1, ∆*PlYY1*-2, and ▲*PlYY1*-2 (Figure 5A,B). Our results demonstrated that deletion of *PlYY1* has not affection on oospore development.

### 3.6. Contributions of PlYY1 to the Pathogenesis of P. litchii

The contribution of *PlYY1* to the virulence of *P. litchii* was investigated by means of pathogenicity assessment. Inoculation of tender litchi leaves and fruits with sporangia suspensions of WT and two mutant strains was carried out, followed by incubation at 25 °C. The lesion area of the leaves and the lesion diameter of the fruits were measured at 48 and 60 h post-inoculation (hpi). The findings revealed a marked reduction in the mutants’ pathogenicity compared to the WT strain.

**Figure 6:**
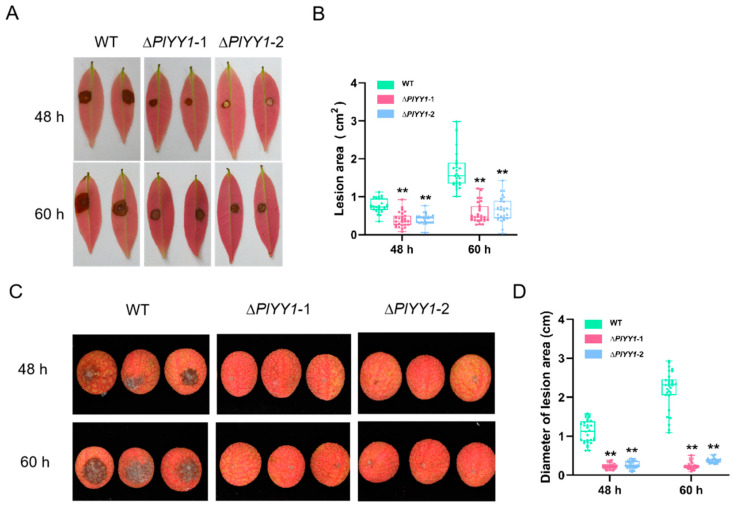
Pathogenicity analysis of WT and mutant strains. (**A**): The areas of lesion on leaves at 48 and 60 h post-inoculation with mycelia plugs from the individual strains. Images showed representative leaves for each instance. (**B**): lesion area of leaves was measured at 48 hpi and 60 hpi. (*n* = 27, ** *p* < 0.01). (**C**): The areas of lesion on fruits at 48 and 60 h post-inoculation with sporangia suspensions of various strains, Images showed representative fruits for each instance. (**D**): lesion area of fruits was measured at 48 hpi and 60 hpi. (*n* = 30, ** *p* < 0.01).

In the leaves assay, the lesion area infected by the WT strain was 0.76 cm^2^ at 48 hpi, while the lesion areas infected by mutant strains ∆*PlYY1*-1 and ∆*PlYY1*-2 were approximately 0.40 and 0.42 cm^2^, respectively. Similarly, at 60 hpi, the lesion area infected by the WT strain increased to 1.71 cm^2^, whereas the lesion areas infected by mutant strains ∆*PlYY1*-1 and ∆*PlYY1*-2 were approximately 0.57 and 0.70 cm^2^ respectively (Figure 6A,B).

In the assay of the fruit, at 48 hpi, the diameter of the disease spot infected by WT was 1.13 cm, while that infected by mutant strains ∆*PlYY1*-1 and ∆*PlYY1*-2 was approximately 0.23 cm and 0.27 cm, respectively. At 60 hpi, the diameter of the disease spot caused by WT increased to 2.18 cm, whereas those caused by mutant strains 1 and 2 were only approximately 0.24 cm and 0.38 cm, respectively (Figure 6C,D).

### 3.7. PlYY1 Promoted the Sensitivity of P. litchii to 6PP

**Figure 7:**
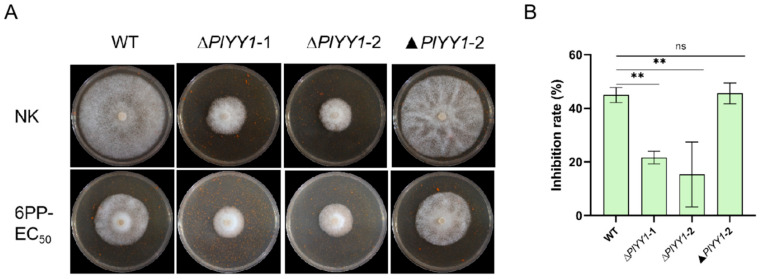
PlYY1 responses to 6PP stress. (**A**): The growth of WT, ▲*PlYY1*-2, ∆*PlYY1*-1, and ∆*PlYY1*-2 on CJA medium without or with 1.0 × EC50 6PP. (**B**): The inhibition rates of 6PP in four strains were calculated by measuring the growth diameters. (** *p* < 0.01, ns represented no significant difference).

Upon comparison of mycelial growth inhibition rate, it was found that In the WT and the complementary strain ▲*PlYY1*-2, the relative inhibition rate was nearly 45.57%; while the two mutant strains exhibited a relatively mild inhibition rate in response to 6PP treatment, approximately 18.47% (Figure 7A,B). This finding suggests that the WT displays greater sensitivity to 6PP. From these observations, we asserted that *PlYY1* possibly functions as a negative regulator of *P. litchii’s* response to 6PP.

## 4. Discussion

According to previous research reports, the TOR pathway plays a crucial role in regulating the vegetative growth and virulence of phytopathogenic fungi [25,36]. TOR and autophagy play a key role in cell growth and virulence-related processes in pathogens, and TOR is one of the negative regulatory factors of autophagy [37]. Comparative transcriptomic analyses revealed significant difference in the expression pattern of four TOR pathway-related genes compared to autophagy-related genes in *P. litchii* strains challenged with 6PP. 

The results indicated that 6PP likely enhances the activities of the TOR pathway by regulating the expression of genes associated with the TOR pathway while suppressing autophagy activity.

Meanwhile, The BlastP methodology was employed to compare the protein sequences of four distinct proteins with those of TOR pathway proteins across multiple model organisms. The results indicated that g12953.t1 protein sequence exhibited a high homology with Cytochrome C and has been identified as a key factor in cell death, specifically in the mediation of cell apoptosis [38], g125.t1 had a high homology with Mitogen-activated protein kinase Spm1 (MAPK), and MAPK signaling pathway is crucial for various cell functions in eukaryotes, such as growth, survival, and stress response [39]. The protein g2677.t1 with high homology to rhoH12, a negative regulator of guanosine triphosphate (GTP) metabolic enzymes in the Ras superfamily, these three genes are known to be associated with essential functions promoter [40]. The PlYY1.t1 had a high homology with the YinYang1 transcription factor, which is known to regulate both the activation and inhibition of transcription for different genes [41].

Moreover, analyses of the potential binding pockets, binding possibilities, and stabilities between 6PP and PlYY1 revealed that the four difference expression level TOR pathway-related proteins can stably bind to the 6PP ligand. These results showed that 6PP mediated TOR activity mainly by regulating TOR pathways-related genes, thereby inhibiting the growth and development of *P. litchii*.

Previous studies showed that YY1 promotes cancer and tumor development in humans. Also, in *Zea mays* (maize), and *Arabidopsis thaliana*, YY1 functions have been linked to vegetative growth [42,43,44,45,46,47]. However, no information regarding the direct and indirect contributions of YY1 in *P. litchii* and other phytopathogenic oomycetes, including *P. litchii*. In this study, we demonstrated using transcriptional activation assays to confirm that the transcription factor YY1 in *P. litchii* binds to regulatory proteins associated with the TOR pathway, but further experiments are needed to confirm this speculation. Genetic deactivation of the *PlYY1* gene triggered significant inhibition in vegetative growth, sporangia production, encystment, zoospore release, and virulence of *P. litchii* without altering oospore production. These findings indicated that *PlYY1* is essential in vegetable growth and asexual reproduction in *P. litchii*. The current observations partly and independently validate previous reports linking the YY1 transcription factor to embryonic (peri-implantation) survival in mice [48] and as a positive regulator of cell proliferation in Humans [49].

Similarly, we demonstrated that 6PP treatments triggered a significant enhancement in the expression pattern of the PlYY1 gene and compromised the sensitivity of *PlYY1* defective strains to 6PP. These observations revealed *PlYY1* as a probable novel target for 6PP. Additionally, we reasoned that 6PP influences vegetative development, cellular differentiation, and pathogenesis in *P. litchii* via PlYY1-mediated regulation of the TOR pathway.

## 5. Conclusions

In the study, we preliminarily explored the response of the TOR pathway to 6PP stress in *P. litchii* by identifying and analyzing TOR pathway-related genes expression, molecular docking, knocking out the *PlYY1* gene, and the sensitivity of the PlYY1 gene to 6PP. These results will help improve our understanding of the mechanism response to 6PP stress and promote more effective disease management strategies in *P. litchii*. However, the function of TOR is complex in eukaryotes and provides clues into the pathological significance of the TOR in *P. litchii*.

## Figures and Tables

**Figure A1:**
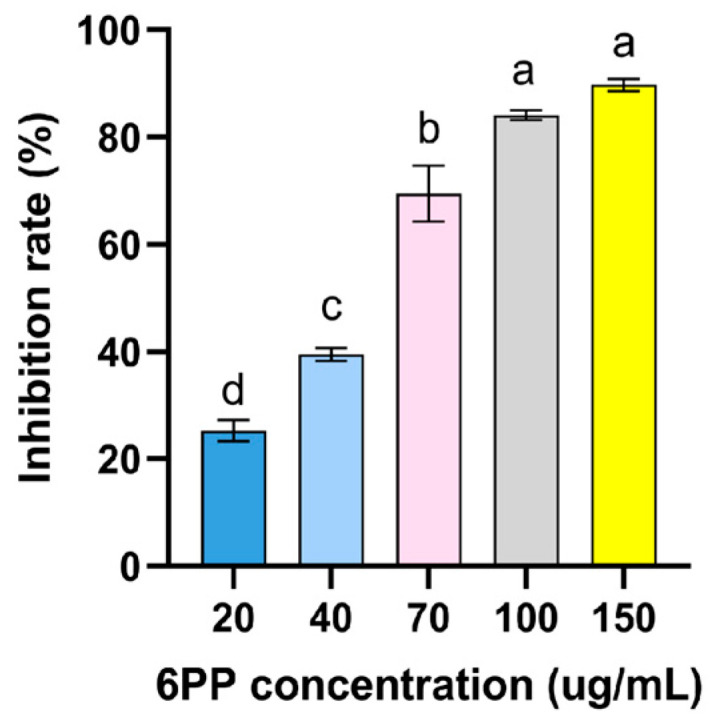
Inhibitory effect of different concentration 6PP on the mycelial growth of *P. litchii*. Due to its volatile nature, 6PP was evaluated its antifungal activity against *P. litchii* by air fumigation. The fumigation device is composed of two petri dish bottoms. One bottom of the petri dish contains *P. litchii* disc (Φ = 5 mm) on PDA medium, and the other bottom of the petri dish contains different concentrations of 6PP (20, 40, 70, 100, and 150 μg/mL), then wrapped with parafilm. fumigation device allows air exchange, but 6PP has no direct contact with *P. litchii*. Colony diameter was measured after 7 d of growth at 25 °C and calculate inhibition rate. The concentration of 6PP inhibiting 50% of mycelial growth (EC50) was estimated to be 43 μg/mL. Data were represented as mean ± standard error (SE) and subjected to analyses of variance (ANOVA) using SPSS ver.19.0 statistical software (SPSS, Chicago, IL, USA). Significant differences among treatments were assessed through Tukey’s test at *p* < 0.05.

## Data Availability

All other study data are included in the article and/or supporting information, further inquiries can be directed to the corresponding author.

## Supplementary Materials

The following supporting information can be downloaded at: https://www.mdpi.com/article/10.3390/jof9080863/s1, Table S1: list of primer pairs used in this study.

## References

[1] Jiang Y., Zhu X., Li Y. (2001). Postharvest control of litchi fruit rot by *Bacillus subtilis*. LWT Food Sci. Technol..

[2] Jiang Y., Wang Y., Song L., Liu H., Lichter A., Kerdchoechuen O., Joyce D., Shi J. (2006). Postharvest characteristics and handling of litchi fruit—An overview. Aust. J. Exp. Agric..

[3] Kong G., Chen Y., Deng Y., Feng D., Jiang L., Wan L., Li M., Jiang Z., Xi P. (2020). The Basic Leucine Zipper Transcription Factor PlBZP32 Associated with the Oxidative Stress Response Is Critical for Pathogenicity of the Lychee Downy Blight Oomycete *Peronophythora litchii*. mSphere.

[4] Wang H., Sun H., Ma J., Stammler G., Zhou M. (2009). Fungicide effectiveness during the various developmental stages of *Peronophythora litchii* in vitro. J. Phytopathol..

[5] Chi P., Pang X., Liu R. (1984). On downy blight of *Litchi chinensis* (Sonn. I). The pathogen and its infection process. Acta. Phytopathol. Sin..

[6] Laugé R., De-Wit P. (1998). Fungal avirulence genes: Structure and possible functions. Fungal. Genet. Biol..

[7] Niazian M., Sabbatini P. (2021). Traditional in vitro strategies for sustainable production of bioactive compounds and manipulation of metabolomic profile in medicinal, aromatic and ornamental plants. Planta.

[8] Demain A. (1992). Microbial secondary metabolism: A new theoretical frontier for academia, a new opportunity for industry. Ciba. Found. Symp..

[9] Collins R., Halim A. (1972). Characterization of the major aroma constituent of the fungus *Trichoderma viride*. J. Agric. Food Chem..

[10] Claydon N., Allan M., Hanson J., Avent A. (1987). Antifungal alkyl pyrones of *Trichoderma harzianum*. Trans. B Mycol. Soc..

[11] Pezet R., Pont V., Tabacch I. (1999). Simple analysis of 6-pentyl-α-pyrone, a major antifungal metabolite of *Trichoderma* spp., useful for testing the antagonistic activity of these fungi. Phytochem. Anal..

[12] Chen L., Cui Y., Yang X., Zhao D., Shen Q. (2012). An antifungal compound from *Trichoderma harzianum* SQR-T037 effectively controls Fusarium wilt of cucumber in continuously cropped soil. Australas. Plant Pathol..

[13] El-Hasan A., Walker F., Schöne J., Buchenauer H. (2007). Antagonistic effect of 6-pentyl-alpha-pyrone produced by *Trichoderma harzianum* toward *Fusarium moniliforme*. J. Plant. Dis. Prot..

[14] Jin X., Guo L., Jin B., Zhu S., Mei X., Wu Q., Liu T., He X. (2020). Inhibitory mechanism of 6-Pentyl-2H-pyran-2-one secreted by *Trichoderma atroviride* T2 against *Cylindrocarpon destructans*. Pestic. Biochem. Physiol..

[15] Tao L., Zhang Y., Li Y., Zhang Z., Chen J. (2020). Antagonistic activity of volatile metabolites from Trichoderma asperellum. Sheng Wu Gong Cheng Xue Bao.

[16] Hunter T. (1995). When is a lipid kinase not a lipid kinase? When it is a protein kinase. Cell.

[17] Saxton R., Sabatini D. (2017). mTOR signaling in growth, metabolism, and disease. Cell.

[18] Gonzalez A., Hall M. (2017). Nutrient sensing and TOR signaling in yeast and mammals. EMBO J..

[19] Loewith R., Jacinto E., Wullschleger S. (2002). Two TOR complexes, only one of which is rapamycin sensitive, have distinct roles in cell growth control. Molecular. Cell.

[20] Wullschleger S., Loewith R., Hall M. (2006). TOR signaling in growth and metabolism. Cell.

[21] Noda T., Ohsumi Y. (1998). Tor, a phosphatidylinositol kinase homologue, controls autophagy in yeast. J. Biol. Chem..

[22] Pattingre S., Espert L., Biard-Piechaczyk M., Codogno-Biochimie P. (2008). Regulation of macroautophagy by mTOR and Beclin 1 complexes. Biochimie.

[23] Beth L., Guido K. (2008). Autophagy in the Pathogenesis of Disease. Cell.

[24] Maiuri M., Galluzzi L., Morselli E., Kepp O., Malik S.A., Kroemer G. (2010). Autophagy regulation by p53. Curr. Opin. Cell Biol..

[25] Yu F., Gu Q., Yun Y., Yin Y., Xu J., Shim W., Ma Z. (2014). The TOR signaling pathway regulates vegetative development and virulence in *Fusarium graminearum*. New Phytol..

[26] Li L., Zhu T., Song Y., Luo X., Feng L., Zhuo F., Li F., Ren M. (2019). Functional characterization of target of rapamycin signaling in *Verticillium dahliae*. Front. Microbiol..

[27] Li L., Zhu T., Song Y., Luo X., Datla R., Ren M. (2021). Target of rapamycin controls hyphal growth and pathogenicity through FoTIP4 in *Fusarium oxysporum*. Mol. Plant Pathol..

[28] Xiong F., Liu M., Zhuo F., Yin H., Deng K., Feng S., Liu Y., Luo X., Feng L., Zhang S. (2019). Hostinduced gene silencing of BcTOR in Botrytis cinerea enhances plant resistance to grey mould. Mol. Plant Pathol..

[29] Gesabel Y., Antonio D., Manuel S. (2022). Constitutive activation of TORC1 signalling attenuates virulence in the cross-kingdom fungal pathogen *Fusarium oxysporum*. Mol. Plant Pathol..

[30] Marroquin-Guzman M., Wilson R. (2015). GATA-Dependent Glutaminolysis Drives Appressorium Formation in *Magnaporthe oryzae* by Suppressing TOR Inhibition of cAMP/PKA Signaling. PLoS Pathog..

[31] Zhang H., Guo L., Li Y., Zhao D., Liu L., Chang W., Zhang K., Zheng Y., Hou J., Fu C. (2022). TOP1α fine-tunes TOR-PLT2 to maintain root tip homeostasis in response to sugars. Nat. Plants.

[32] Blenis J. (2017). TOR, the gateway to cellular metabolism, cell growth, and disease. Cell.

[33] Kraushaar D., Jin W., Maunakea A., Abraham B., Ha M., Zhao K. (2013). Erratum to: Genome wide incorporation dynamics reveal distinct categories of turnover for the histone variant H3.3. Genome Biol..

[34] Wang Z., Ke Q., Tao K., Li Q., Xia Y., Bao J., Chen Q. (2022). Activity and Point Mutation G699V in PcoORP1 Confer Resistance to Oxathiapiprolin in *Phytophthora colocasiae* Field Isolates. J. Agric. Food Chem..

[35] Liu X., Dong J., Liao J., Tian L., Qiu H., Wu T., Ge F., Zhu J., Shi L., Jiang A. (2020). Establishment of CRISPR/Cas9 Genome Editing System in *Peronophythora litchii*. J. Fungi Res..

[36] Menand B., Meyer C., Robaglia C. (2004). Plant growth and the TOR pathway. Curr. Top. Microbiol. Immunol..

[37] Huang C., Li L., Wang L., Bao J., Zhang X., Yan J., Wu J., Cao N., Wang J., Zhao L. (2022). The Amino Acid Permease MoGap1 Regulates TOR Activity and Autophagy in *Magnaporthe oryzae*. Int. J. Mol. Sci..

[38] Liu X., Kim C., Yang J., Jemmerson R., Wang X. (1996). Induction of apoptotic program in cell-free extracts: Requirement for dATP and cytochrome c. Cell.

[39] Roskoski R. (2012). erk1/2 map kinases: Structure, function, and regulation. Pharmacol. Res..

[40] Cox A., Der C. (2003). the dark side of ras: Regulation of apoptosis. Oncogene.

[41] Verheul T., Hijfte L., Perenthaler E., Barakat T. (2020). The Why of YY1: Mechanisms of Transcriptional Regulation by Yin Yang 1. Front. Cell Dev. Biol..

[42] Riggs K., Saleque S., Wong K., Merrell K., Lee J., Shi Y., Calame K. (1993). Yin-yang 1 activates the c-myc promoter. Mol. Cell Biol..

[43] Shi Y., Seto E., Chang L., Shenk T. (1991). Transcriptional repression by YY1, a human GLI-Krüippel-related protein, and relief of repression by adenovirus E1A protein. Cell.

[44] Petkova V., Romanowski M., Sulijoadikusumo I., Rohne D., Kang P., Shenk T., Usheva A. (2001). Interaction between YY1 and the retinoblastoma protein. Regulation of cell cycle progression in differentiated cells. Biol. Chem..

[45] Wang H., Hertlein E., Bakkar N., Sun H., Acharyya S., Wang J., Carathers M., Davuluri R., Guttridge D. (2007). NF-kappaB regulation of YY1 inhibits skeletal myogenesis through transcriptional silencing of myofibrillar genes. Mol. Cell Biol..

[46] Xu T., Purcel M., Zucchi P., Helentjaris T., Bogorad L. (2001). TRM1, a YY1-like suppressor of rbcS-m3 expression in maize mesophyll cells. Proc. Natl. Acad. Sci. USA.

[47] Li T., Wu X., Li H., Song J., Liu J. (2016). A Dual-Function Transcription Factor, AtYY1, Is a Novel Negative Regulator of the *Arabidopsis* ABA Response Network. Mol. Plant.

[48] Affar E., Gay F., Shi Y., Liu H., Huarte M., Wu S., Collins T., Li E., Shi Y. (2006). Essential dosage dependent functions of the transcription factor yin yang 1 in late embryonic development and cell cycle progression. Mol. Cell Biol..

[49] Taguchi S., Kawachi Y., Ishitsuka Y., Fujisawa Y., Furuta J., Nakamura Y., Xu X., Ikebe D., Kato M., Otsuka F. (2011). Overexpression of the transcription factor YinYang-1 suppresses differentiation of HaCaT cells in three-dimensional cell culture. J. Investig. Dermatol..

