# Supplementary material for: A New Insight into 6-Pentyl-2H-pyran-2-one against Peronophythora litchii via TOR Pathway
Source: J Fungi (Basel). 2023 Aug 21;9(8):863. doi: 10.3390/jof9080863 (PMC10515317; doi:10.3390/jof9080863)
Supplement: Supplementary file 1 [file jof-09-00863-s001.zip › Primers-0828.pdf]

Table S1. List of primer pairs used in this study

Primers	Sequence (5'-----3')	function
pGBKT7-*PlYY1*-F	CGGAATTCATGATGTCGCTGCTCGCTC	pGBKT7-*PlYY1* construct F primer
pGBKT7-*PlYY1*-R	GGGATCCCTACACGTTCTTAGTGAGCTCGAAC	pGBKT7-*PlYY1* construct R primer
PlYY1-sg1F	CTAGCGCACACCTGATGAGTCCGTGAGGACGAAACGAGTAAGCTCGTCGTGTGCGAGGAACCCGGTTG	F primer of sgRNA1
PlYY1-sg1R	AAACCAACCGGGTTCCTCGCACACGACGAGCTTACTCGTTTCGTCCTCACGGACTCATCAGGTGTGCG	R primer of sgRNA1
PlYY1-sg2F	CTAGCGTGGTGCTGATGAGTCCGTGAGGACGAAACGAGTAAGCTCGTCCACCACATCAAGATCCACAC	F primer of sgRNA2
PlYY1-sg2R	AAACGTGTGGATCTTGATGTGGTGGACGAGCTTACTCGTTTCGTCCTCACGGACTCATCAGCACCACG	R primer of sgRNA2
*PlYY1*AF	CCCCTCGAGGTCGACGGTATACATGATGTGGAGCGCGTC	Up stream F primer of *PlYY1* / Complement F primer
*PlYY1*AR	GCTTTTTCATGGTGGCGTTGAGGTATCC	Up stream R primer of *PlYY1*
*PlYY1*hphF	CAACGCCACCATGAAAAAGCCTGAACTCAC	1) Donor DNA F primer. 2)F prier to verifying whether donar DNA *HPH* and down stream sequence

		can be amplified
*PlYY1*hphR	TCCTACACGCCTATTCCTTTGCCCTCGG	Donor DNA R primer
*PlYY1*BF	AAAGGAATAGGCGTGTAGGATATTGAGTATTTC	Down stream F primer of *PlYY1*
*PlYY1*BR	CGGCCGCTCTAGAACTAGTGCATACATGTACATGAATGGTG	1)Down stream R primer of *PlYY1*. 2)F primer to verifying whether donar DNA *HPH* and down stream sequence can be amplified. 3)Complement F primer
A1-F primer	CTCGACCACCACATCAAGATCCACA	verifying whether *PlYY1* can be amplified
A1-R primer	TGAGATACACCCGCCACGCTG	
